# Activation of c-Myc and Cyclin D1 by JCV T-Antigen and β-Catenin in Colon Cancer

**DOI:** 10.1371/journal.pone.0106257

**Published:** 2014-09-17

**Authors:** Michael J. Ripple, Amanda Parker Struckhoff, Jimena Trillo-Tinoco, Li Li, David A. Margolin, Robin McGoey, Luis Del Valle

**Affiliations:** 1 Stanley S. Scott Cancer Center, Department of Medicine, Louisiana State University Health Sciences Center, New Orleans, Louisiana, United States of America; 2 Department of Genetics, Louisiana State University Health Sciences Center, New Orleans, Louisiana, United States of America; 3 Institute for Translational Research, Ochsner Clinic Foundation, New Orleans, Louisiana, United States of America; 4 Department of Colorectal Surgery, Ochsner Clinic Foundation, New Orleans, Louisiana, United States of America; 5 Department of Pathology, Louisiana State University Health Sciences Center, New Orleans, Louisiana, United States of America; University of Texas Medical Branch, United States of America

## Abstract

During the last decade, mounting evidence has implicated the human neurotropic virus JC virus in the pathology of colon cancer. However, the mechanisms of JC virus-mediated oncogenesis are still not fully determined. One candidate to mediate these effects is the viral early transcriptional product T-Antigen, which has the ability to inactivate cell cycle regulatory proteins such as p53. In medulloblastomas, T-Antigen has been shown to bind the Wnt signaling pathway protein β-catenin; however, the effects of this interaction on downstream cell cycle regulatory proteins remain unknown. In light of these observations, we investigated the association of T-Antigen and nuclear β-catenin in colon cancer cases and the effects of this complex in the activation of the transcription and cell cycle regulators c-Myc and Cyclin D1 *in vitro*. Gene amplification demonstrated the presence of viral sequences in 82.4% of cases and we detected expression of T-Antigen in 64.6% of cases by immunohistochemistry. Further, we found that T-Antigen and β-catenin co-localized in the nuclei of tumor cells and we confirmed the physical binding between these two proteins *in vitro*. The nuclear presence of T-Antigen and β-catenin resulted in the significant enhancement of TCF-dependent promoter activity and activation of the β-catenin downstream targets, c-Myc and Cyclin D1. These observations provide further evidence for a role of JCV T-Antigen in the dysregulation of the Wnt signaling pathway and in the pathogenesis of colon cancer.

## Introduction

Colorectal carcinoma (CRC) remains a serious health problem around the world despite advances in diagnosis and treatment. It ranks third in overall cancer incidence and is the second most common cause of cancer mortality in the United States where there are an estimated 140,000 new cases and 50,000 CRC-related deaths each year [1]. The risk of developing sporadic colon cancer in the general population is 5%, but this incidence increases with age due to the accumulation of genetic and epigenetic alterations [2]. Some of these alterations can be caused by environmental factors, including viruses, which have been implicated in several other types of cancer. Several independent studies have established that approximately 20% of all cancers are caused by infectious agents [3], [4]. One such agent is the human neurotropic JC virus (JCV), a member of the *Polyomaviridiae* family, which also includes SV-40, BK virus (BKV) and the newly discovered Merkel Cell Polyomavirus (MCPyV), found to be clonally integrated in a high percentage of Merkel cell carcinomas. JCV has been established as the causative agent of Progressive Multifocal Leukoencephalopathy (PML), a demyelinating disease affecting HIV patients [5], and more recently in patients undergoing immunomodulatory therapy [6]. During the last decade, several laboratories have demonstrated that JCV is associated with various human cancers, including brain tumors [7], [8], lung cancer [9], and malignancies of the upper and lower digestive tracts [10]–[12].

JCV is a ubiquitous virus that infects a large proportion of the general population. According to sero-epidemiological studies, primary infection with JCV occurs in early childhood and over 70–90% of adult individuals are positive for anti-JCV antibodies [13], [14]. Although primary infection is asymptomatic, the proposed route of transmission is via the respiratory tract, after which JCV establishes a latent infection in the kidney. In approximately 30% of healthy seropositive individuals, JCV is shed in the urine under normal conditions [15], [16]. As a result, intact JCV particles have been isolated from samples of raw urban sewage from around the world [17], [18], which together with the finding of viral DNA in epithelial cells from the upper and lower digestive tracts [19], suggests an additional point of entry for the virus through the GI tract via exposure to contaminated water or food. Importantly, Bofill-Mas, *et al* showed that viral particles isolated from sewage remained intact after treatment at a pH of 1 for 30 minutes, indicating that ingestion of contaminated water or food may be sufficient for JCV infection of the gastrointestinal tract [20]. Many studies since, have demonstrated the presence of JCV genomic sequences and the expression of T-Antigen in tissues from gastrointestinal tumors, including esophageal carcinoma [11], gastric carcinoma [12], [21], [22], sporadic adenomatous polyps [23], and colorectal adenocarcinomas [10], [24]–[30].

The JCV genome is approximately 5.2 kb in size and can be divided into three regions: the early viral genomic region (EVGR), the late viral genomic region (LVGR), and the non-coding control region (NCCR), which contains the origin of replication, and is located between early and late genomic regions. The EVGR encodes a powerful oncogenic protein, large T-Antigen, while the LVGR encodes the viral capsid proteins VP1, VP2 and VP3, as well as the Agnoprotein, a small accessory product [31]. The current model of JCV-mediated oncogenesis involves the integration of portions of the JCV genome, particularly the T-Antigen gene, which is proposed to increase the risk of developing cancer due to the subsequent expression of T-Antigen. In support of this model, Theodoropoulos *et al* showed that the JCV viral load was 10- to 50- fold higher in colon adenomas and adenocarcinomas compared to matched normal tissue [32]. Additionally, Coelho *et al* recently demonstrated that CRC tissue has a significantly higher relative frequency of JCV genomic sequences than adjacent normal mucosa and distant normal mucosa [33].

The transforming abilities of JCV T-Antigen *in vitro* are well known [34]. *In vivo* T-Antigen causes a variety of Central Nervous System tumors when injected into the brain of rodents and non-human primates, ranging from medulloblastomas to glial tumors [35]–[37]. Transgenic mice that express JCV T-Antigen from the NCCR develop tumors of neuroectodermal origin, including medulloblastomas, glial tumors, and malignant peripheral nerve sheath tumors [38]–[40]. This transformation process occurs through the interaction of T-Antigen with key cell cycle regulatory proteins, including p53, Rb, and the central signaling molecule in the Wnt signaling pathway, β-catenin [41]–[43].

β-catenin is often dysregulated in colon cancer [44], [45]. Levels of β-catenin are tightly controlled by its destruction complex, consisting of the proteins APC, Axin, GSK3, and CK1, which mark it for ubiquitination. Mutations in the components of the destruction complex can result in aberrant nuclear localization of β-catenin, a well-established mechanism of carcinogenesis in colorectal cancer [44], [46] which leads to the activation of Wnt pathway target genes in the absence of Wnt signaling and results in unchecked cellular proliferation [2]. While the Wnt pathway is activated by genetic mutations in over 90% of colon cancer cases [47], nuclear β-catenin expression is only detected in approximately 50% of cases [48], suggesting that there are additional factors that contribute to the nuclear localization of β-catenin in colon cancer. It has been demonstrated that JCV T-Antigen has the ability to bind β-catenin through an interaction with the C-terminus of β-catenin [41], and that T-Antigen can stabilize β-catenin in glioblastoma cells [49]. In addition, T-Antigen has a classical nuclear localization signal (NLS) and is strongly expressed mainly in the nuclei of neoplastic cells [10], [42], [50]. Therefore, we sought to determine the effect of JCV T-Antigen on the nuclear localization of β-catenin and the role of this complex in the activation of β-catenin target genes as a mechanism in the development and progression of colon cancer.

In the current study, we measure the prevalence of T-Antigen genomic sequences and protein expression in human colon cancer tissues using a collection of well-characterized biopsy samples. Importantly, we demonstrate an association of T-Antigen and nuclear β-catenin in human colon cancer samples and elaborate on this association by elucidating the molecular effects of this interaction in the dysregulation of the Wnt signaling cascade and the activation of β-catenin targets using colon cancer cell lines.

## Materials and Methods

### Clinical Samples

A total of 113 de-identified paraffin-embedded samples of colorectal carcinoma and pre-neoplastic polyps were collected from the pathology archives of the following institutions: LSUHSC-New Orleans (34 samples) and the Ochsner Clinic (79 samples). The tumors were were histologically graded and immunohistochemically characterized according to the WHO Classification of Tumors of the Gastrointestinal Tract.

### DNA Extraction and Analysis

A dedicated microtome was used to section the paraffin blocks in order to avoid contamination. Furthermore, the block and blade holder were periodically autoclaved, and a new, disposable blade was used for each specimen. DNA was extracted from approximately 10 sections of 10 µm in thickness from each of the tissue samples by using the QIAamp Tissue Kit, according to the manufacturer’s instructions (Qiagen). PCR amplification of the T-Antigen gene was performed using PEP1 and PEP2 primers (nucleotides 4255–4272 and 4408–4427, respectively), which amplify sequences in the N-terminal region of JCV T-Antigen (PEP1 sequence: AGTCTTTAGGGTCTTCTACC; PEP2 sequence: AGGTGCCAACCTATGGAACA). For amplification of the Control Region (CR), we used the NCRR1 and NCRR2 primers, corresponding to nucleotides 4993–5004 and 258–279, respectively (NCRR1 sequence: GCAAAAAAGCGAAAAACAAGGG; NCRR2 sequence: CAGAAGCCTTACGTGACAGCTGG). Amplification was carried out on 250 ng of template DNA with the FailSafe PCR system (Epicentre) in a total volume of 25 µl. PCR was run at the following conditions: denaturation at 95°C for 10 min, followed by 30 cycles of denaturation at 95°C for 15 s, annealing at 62°C for 30 s, and extension at 72°C for 30 s. As a termination step, the extension time of the last cycle was increased to 7 min. Samples amplified in the absence of template DNA served as a negative control, whereas DNA extracted from T-Antigen positive BSB8 cells was used as a positive control for each procedure. Southern blot was performed by resolving 10 µl of each PCR reaction on a 2% agarose gel. The gel was then treated for 15 min each with 0.2 M HCl for depurination, 1.5 M NaCl/0.5 M NaOH for denaturation, and 1.5 M NaCl/0.5 M Tris-HCl (pH 7.4) for neutralization, followed by transfer of the amplified fragments from the gel to nylon membranes (Hybond-N; Amersham). The membranes were then pre-hybridized for 1 h in EasyHyb solution (Roche), followed by hybridization in the same solution containing 2 µg DIG-labeled oligonucleotide specific for JCV T-Antigen (nucleotides 4304–4405) overnight. Primers used for synthesis of DIG labeled probe are PEP INT 1 (TTGGGATCCTGTGTTTTCATC) and PEP INT 2 (AATGGGAATCCTGGTGGAAT). For the CR probe we utilized primers from nucleotides 62–81. Probes were synthesized using the DIG DNA Labeling and Detection Kit (Roche). Membranes were hybridized overnight, washed, and developed using the DIG Wash and Block Buffer Set following the manufacturer’s instructions (Roche).

DNA sequencing of the control region was performed using ABI Prism 3730x/DNA analyzer. For amplification of DNA from the housekeeping gene, GAPDH, the following primers were used:

5′ TTC TCC CCATTC CGT CTT CC 3′ and 3′ GTA CAT GGT ATT CAC CAC CC 5′.

### Histological and Immunohistochemical Analysis

Paraffin-embedded tissue previously fixed in 10% buffered formalin was sectioned at 4-**µ**m thickness and mounted onto charged slides. Sections were placed in an oven at 65°C to melt the paraffin and then deparaffinized in three changes of xylene for 30 min each. Sections were then rehydrated through a graded series of alcohols up to water, and non-enzymatic antigen retrieval was performed in 0.01 M sodium citrate (pH 6.0) at 97°C in a vacuum oven for 35 min. After a cooling period of 25 min, sections were rinsed with PBS, and endogenous peroxidase was quenched by incubating the slides in methanol/3% H_2_O_2_ for 30 min at room temperature. Sections were blocked in 2% horse serum and incubated overnight with primary antibodies at room temperature in a humidified chamber. Antibodies used to detect viral proteins included a mouse monoclonal antibody against SV40 large T-Antigen that cross-reacts with JCV T-Antigen (clone pAb416, 1∶100 dilution; Calbiochem) and a mouse monoclonal anti-β-catenin antibody (clone E-5, 1∶100 dilution; Santa Cruz Biotechnology). After rinsing the sections in PBS, the slides were incubated for 1 hour at room temperature with biotinylated anti-mouse secondary antibodies and then were rinsed in PBS. The tissue was subsequently incubated with avidin-biotin-peroxidase complexes for 1 hour at room temperature according to the manufacturer’s instructions (Vector Laboratories), and finally, the sections were developed with a 3,3′-diaminobenzidine substrate (Sigma), counterstained with Hematoxylin, and coverslipped with Permount (Fisher). To assess the fraction of immunolabeled cells in specimens from each patient case, the labeling index defined as the percentage of positive cells of the total number of tumor cells counted was determined.

### Double-labeling Immunofluorescence

For immunofluorescence and double labeling of paraffin embedded sections, deparaffinization, antigen retrieval, endogenous peroxidase quenching and blocking were performed as described above. For immunofluorescent double labeling of HCT116 cells in culture, the cells were washed with PBS, fixed in cold acetone, and blocked in PBS containing 5% horse serum and 0.1% BSA for 2 h. Sections or cells were then incubated with mouse anti-T-Antigen antibody (clone pAb416, 1∶100 dilution, Oncogene Science) and rabbit anti-β-catenin antibody (clone D13A1, 1∶100 dilution Cell Signaling) for 16 hours followed by washing in PBS and incubation in anti-mouse Rhodamine antibody and anti-rabbit Fluorescein antibody (1∶200 dilution; Vector Laboratories). Finally, sections were washed in PBS and mounted in aqueous mounting media with DAPI (Vector Laboratories), and visualized in a confocal microscope (Olympus FV1000).

### Transient Transfection and Luciferase constructs

HCT116 cells were kindly provided by Dr. Hamid Boulares. Cells were transfected using Xtreme Gene-HP (Roche). The following constructs were used for luciferase assays: M72 Super 16x TOPflash reporter construct: Addgene plasmid 17165; M51 Super 8x FOPflash: Addgene plasmid 12457; c-Myc promoter: Addgene plasmid 16595; Cyclin D1 promoter: Addgene plasmid 32727. These reporter constructs were transfected alone or together with pcDNA-T-Antigen and/or pcDNA-β-catenin and assayed for luciferase activity using a Luciferase Assay System (Promega) at 24 hours post-transfection.

### Protein Extraction, Co-Immunoprecipitation and Analysis

Cytoplasmic and nuclear protein extracts from HCT116 cells were collected in using cytoplasmic lysis buffer and nuclear lysis buffer as previously described [51]. Briefly, cells were trypsinized, pelleted at 2000 g for 5 minutes, and re-suspended in 500 ul cytoplasmic lysis buffer for 10 minutes on ice. Following lysis of the cell membrane, nuclei were pelleted at 14,000 g for 10 minutes. Nuclei were washed once in cytoplasmic lysis buffer, pelleted and lysed in 250 ul nuclear lysis buffer for 20 minutes at 4°C with shaking. 500 µg total protein extract was incubated with 10 µg anti-T-Antigen or anti-β-catenin antibody overnight at 4°C with shaking. Antigen-antibody complexes were then immunoprecipitated with Protein A/G magnetic beads (Pierce), washed in HNTG buffer (20 mM HEPES, 150 mM NaCl, 10% glycerol, 0.1% Triton X-100), denatured and analyzed by SDS-PAGE.

### Ethics Statement

This study was performed in strict accordance to Louisiana State University’s Institutional Review Board guidelines and approval. All 113 paraffin-embedded human samples of colorectal carcinomas and pre-neoplastic polyps, which were collected from the pathology archives of LSUHSC New Orleans (34 samples) and the Ochsner Clinic (79 samples). The Louisiana State University Institutional Review Board (IRB) waived the need for written consent, as the samples are archival, de-identified and follow under the NIH guidelines for exemption as tissue that would be discarded and cannot be traced back to patients (IRB Protocol #8077).

## Results

### Expression of T-Antigen and β-catenin in colon cancer samples

**Figure 1:**
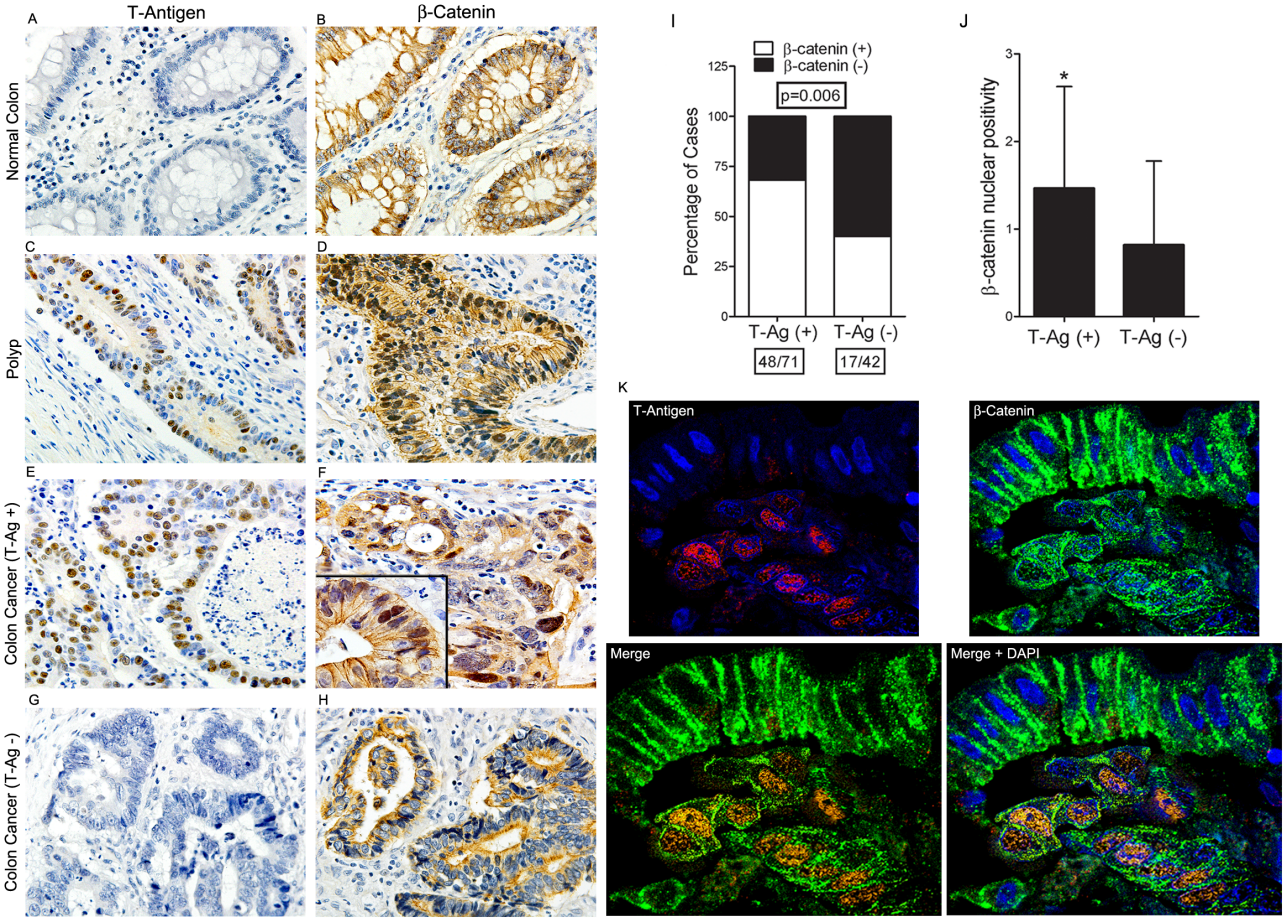
T-Antigen and β-catenin expression in colon cancer cases. Immunohistochemistry in areas of normal colonic epithelium is negative for T-Antigen, while β-catenin is expressed in the cytoplasm and membrane (Panels and B). T-Antigen expression is found in the nuclei of epithelial cells in polyps and neoplastic cells in areas of invasive tumor (Panels C and E, respectively). Consecutive sections of the same cases show that β-catenin is now located to the cytoplasm and nuclei of epithelial and tumor cells (Panels D and F respectively). In T-Antigen negative cases however, β-catenin remains in the cytoplasm (Panels G and H). Statistical analysis reveals that 67.6% of T-Antigen positive colon cancer cases express nuclear β-catenin, while only 40.4% of T-Antigen negative cases express nuclear β-catenin (G; p = 0.006) (Panel I). The relative percentage of nuclei positive for β-catenin in a section was scored on a scale of 0–4. T-Antigen positive cases showed significantly more nuclei positive for β-catenin than T-Antigen negative samples (F; *p<0.05) (Panel J). Double labeling immunofluorescence demonstrates the co-localization of T-Antigen (Rhodamine) and β-catenin (Fluorescein) in the nuclei of neoplastic cells in a pocket of neoplastic cells underneath an epithelial lining of T-Antigen negative cells, in which β-catenin remains cytoplamic (Panel K). Original magnification of all immunohistochemistry panels is 600x, and immunofluorescence is 1000x.

The early transcriptional product of JCV, T-Antigen, is a well-known potent oncogenic protein, which has been detected in a variety of tumors. In order to establish the presence of JCV T-Antigen, we performed immunohistochemical experiments in a total of 113 formalin-fixed, paraffin embedded cases of colon cancer from 2 of New Orleans largest medical institutions. The majority of these samples contained normal mucosa, pre-neoplastic polyps and both *in situ* and invasive carcinoma. We found expression of T-Antigen by immunohistochemistry in the nuclei of tumor cells in 73 of the samples (64.6%). Interestingly, T-Antigen was also present in the nuclei of epithelial cells from pre-neoplastic polyps, but completely absent in the normal colonic epithelium (Figure 1, left panels).

Previously, we have demonstrated that in medulloblastomas, T-Antigen is capable of physically bind and translocate β-catenin, the central molecule of the Wnt signaling pathway, into the nucleus [45], [52]. To determine whether β-catenin is present and associated with T-Antigen in colon cancer, we performed immunohistochemistry in the same 113 samples and analyzed the expression of nuclear β-catenin in T-Antigen positive and negative groups. We found that of the 71 total T-Antigen positive colon cancer samples, 48 showed nuclear expression of β-catenin (67.6%), while only 17/42 (40.5%) of T-Antigen negative samples were positive for nuclear β-catenin (p≤0.005, Figure 1I). In all T-Antigen negative cases, β-catenin remained in the cytoplasm. Interestingly, the same observations were present in the polyps, where nuclear β-catenin correlated with expression of T-Antigen. In the normal mucosa, β-catenin remained localized to its usual location in the cytoplasm and membrane. β-catenin nuclear positive cells were scored on a scale of 0–4, with a score of 0 indicating that ∼5% or fewer cells and a score of 4 indicating ∼75% or more cells with nuclear β-catenin. T-Antigen positive samples had an average score of 1.463 (SD 1.164) for nuclear β-catenin, while T-Antigen negative samples had an average score of 0.8182 (SD 0.9580; p≤0.05, Figure 1J).

Finally, we performed double labeling immunofluorescence in a positive case that contained the three areas (normal colon, polyp and cancer) and found that in the normal colon, where T-Antigen is negative, β-catenin is expressed in the cytoplasm; however, in polyps and neoplastic cells, that express T-Antigen, β-catenin is co-localizing with the JCV oncoprotein in the nuclear compartment. Figure 1, Panel K shows an area that contains normal epithelium at the top, with no expression of T-Antigen and cytoplasmic β-catenin, while an underlying pocket of neoplastic cells that robustly express T-Antigen show the nuclear co-localization of β-catenin.

### Detection of JC virus T-Antigen genomic sequences in colorectal cancer samples

**Figure 2:**
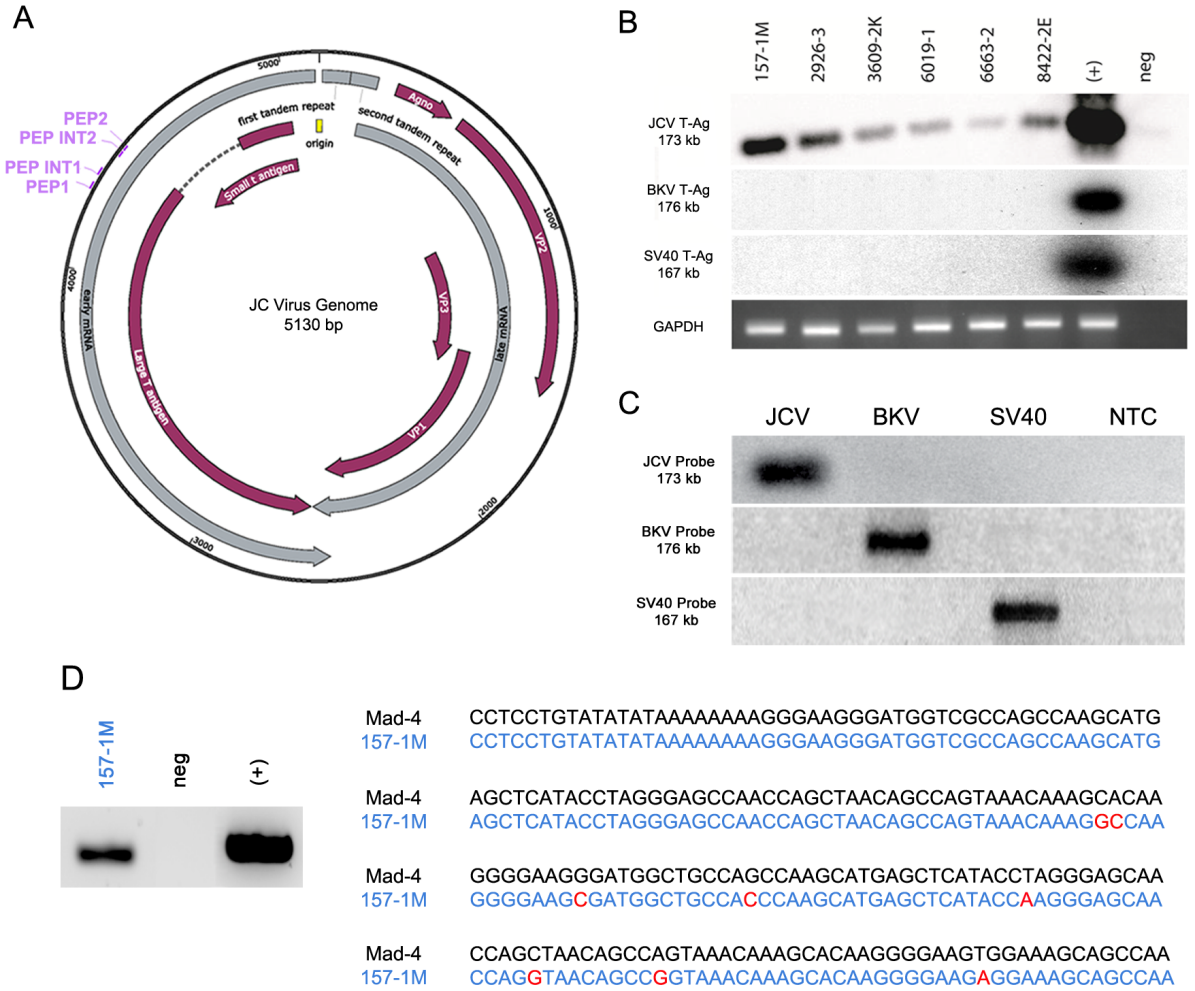
Amplification of JCV T-Antigen genome in colon cancer samples. A schematic representation of the Mad-1 strain of the JCV genomic structure showing viral genes in red, early and late transcripts in grey, and the locations of primers used for T-Antigen amplification (PEP1 and PEP2) and Southern blot probe synthesis (PEP INT1 and PEP INT2) (Panel A). Genomic sequences of the viral early region, which encodes T-Antigen, were detected in 28 of 34 selected samples. A representative Southern blot is presented, with case numbers above each lane. Negative results were observed when the same cases were tested with probes for BKV and SV40. Negative controls show results from reactions containing no DNA and positive control represents gene amplification using pBJC, a plasmid containing JCV DNA as a template or plasmids containing BKV and SV40 T-Antigens. PCR for GAPDH and agarose electrophoresis was used to confirm the presence of intact genomic DNA in all cases. (Panel B). Southern blots of plasmids containing the T-Antigen of JCV, BKV and SV40 were tested with specific probes for each polyomavirus, demonstrating the specificity of the probes (Panel C). A representative Southern blot of PCR amplification of the control regulatory region is shown (Panel D, left). Sequencing revealed the presence of Mad-1 and Mad-4 strains with point mutations in the different samples. A representative sequence is shown in blue, underneath the known sequence of the Mad-4 CR region (black); point mutations are highlighted in red (Panel D, right).

To confirm the presence of the T-Antigen gene, we performed PCR amplification followed by Southern blot using a highly specific and sensitive probe for JCV T-Antigen in a selected number of 34 colon cancer cases that contained high-quality DNA. A schematic representation of the JCV genome is shown, containing the location of primers for Southern blot amplification and probe synthesis (Figure 2A). The region of probe recognition in the JCV genome (base pairs 4304–4405) is highly variable between JCV, BKV, and SV40, with 20 nucleotide mismatches between JCV and BK virus, and 33 nucleotide mismatches between JCV and SV40 in that region. While the PEP1 and PEP2 primers amplify both JCV and BKV T-Antigen our probe recognizing the region between the PEP1 and PEP2 primers is highly specific for JCV and does not bind to BKV or SV40 [53].

We amplified genomic sequences from the T-Antigen coding region in 28 samples of the 34 samples (82.4%), 21 of which demonstrated T-Antigen protein expression by immunohistochemistry. In only six cases we found the presence of viral DNA but no protein expression, and none of the cases had T-Antigen expression in the absence of viral genomic sequences. We tested the amplified sequences with specific probes for BKV and SV40, resulting negative in all cases. High quality genomic DNA was confirmed in each sample by PCR for GAPDH. (Figure 2B). Finally, we also tested our probes with plasmids containing T-Antigens from JCV, BKV an SV40, corroborating the specificity of our probes, and suggesting that the immunohistochemistry reveals the T-Antigen of JCV and not the one from the other polyomaviruses (Figure 2C).

Next, we performed PCR experiments with primers and probes specific for the regulatory region of JCV. We amplified viral sequences in 14 of the 34 cases. Figure 2D shows a representative Southern blot containing one of these cases, which was also positive for the T-Antigen region. Sequencing of these amplicons revealed that Mad-1 and Mad-4 were the two detected stains of JCV. However all of these sequences contained individual point mutations, different from each other, suggesting that these are indeed unique and discarding the possibility of laboratory contamination. A representative sequence is shown in blue, underneath the know sequence of the Mad 4 strain of JCV in black. Point mutations are highlighted in red (Figure 2D). In addition, a detailed summary of the cases and the results of PCR amplification and sequencing, as well as immunohistochemistry is presented in Table 1.

**Table 1:** Molecular and Immunohistochemical analysis of colon cancer cases.

NO.	DIAGNOSIS	AGE	GENDER	LOCATION	PCR		IHC	
					CR	T-Antigen	T-Antigen	β-Catenin
1	Adenocarcinoma+Villous Polyp	62 y/o	Female	Right Colon	Mad-1	+	++	++n/++c
2	AdenoCarcinoma	60 y/o	Female	Sigmoid	−	+	+	+n/++c
3	Mucinous Adenocarcinoma	60 y/o	Female	Rectum	Mad-4	+	−	− n/−c
4	Adenocarcinoma	57 y/o	Male	Cecum	−	+	+	+n/+c
5	AdenoCarcinoma+Villous Polyp	54 y/o	Male	Sigmoid	Mad-1	+	++++	++n/++c
6	Adenocarcinoma	64 y/o	Male	Sigmoid	Mad-4	+	+	+n/++c
7	Mucinous Adenocarcinoma+Villous Polyp	61 y/o	Female	Rectum	−	+	+	+n/+c
8	Adenocarcinoma	40 y/o	Female	Sigmoid	−	+	+	+n/+++c
9	Villous Polyp	57 y/o	Female	Rectum	Mad-4	+	+++	+++n/++c
10	Adenocarcinoma	64 y/o	Male	Cecum	−	−	−	− n/+++c
11	Adenocarcinoma+Villous Polyp	53 y/o	Female	Rectosigmoid	−	−	−	− n/+++c
12	Villous Polyp	53 y/o	Female	Sigmoid	−	−	−	− n/++c
13	Adenocarcinoma+Villous Polyp	71 y/o	Male	Sigmoid	−	+	++	++n/+c
14	Adenocarcinoma	58 y/o	Female	Sigmoid	Mad-1	+	++++	+++n/++c
15	Adenocarcinoma	47 y/o	Male	Rectosigmoid	−	−	−	− n/+c
16	Mucinous Adenocarcinoma	71 y/o	Male	Rectum	Mad-4	+	+	+n/+c
17	Adenocarcinoma+Villous Polyp	59 y/o	Female	Sigmoid	−	+	−	− n/++c
18	Adenocarcinoma	71 y/o	Female	Sigmoid	−	+	−	− n/++c
19	Adenocarcinoma	58 y/o	Female	Sigmoid	−	+	−	− n/+c
20	Adenocarcinoma+Villous Polyp	52 y/o	Female	Right Colon	Mad-1	+	++	++n/++c
21	Adenocarcinoma+Villous Polyp	59 y/o	Female	Cecum	−	+	−	− n/− c
22	Villous Polyp	63 y/o	Male	Cecum	Mad-4	+	+	− n/++c
23	Adenocarcinoma+Villous Polyp	57 y/o	Female	Splenic Flexure	−	+	−	− n/++c
24	Adenocarcinoma	57 y/o	Female	Cecum	−	−	−	− n/+ c
25	Adenocarcinoma	76 y/o	Female	Sigmoid	−	+	−	− n/++ c
26	Adenocarcinoma+Villous Polyp	54 y/o	Male	Sigmoid	Mad-4	+	+	+n/+ c
27	Adenocarcinoma	51 y/o	Female	Rectosigmoid	Mad-1	+	+++	+++n/++c
28	Adenocarcinoma	52 y/o	Male	Rectum	Mad-4	+	+++	++n/+++c
29	Adenocarcinoma+Villous Polyp	52 y/o	Male	Rectosigmoid	−	+	+++	+n/+c
30	Adenocarcinoma+Villous Polyp	60 y/o	Male	Left Colon	Mad-4	+	+++	++n/++c
31	Adenocarcinoma	71 y/o	Female	Rectosigmoid	−	+	++	++n/+++c
32	Adenocarcinoma+Villous Polyp	53 y/o	Male	Right Colon	−	+	+	+n/++c
33	Adenocarcinoma	35 y/o	Female	Left Colon	Mad-4	+	+++	+++n/++c
34	Adenocarcinoma	58 y/o	Male	Sigmoid	−	−	−	− n/− c

Diagnosis of the tumors is based on the World Health Organization Classification of Tumours of the Digestive System.

Age of the patient at the time of surgical resection is shown. M = Male; F = Female. PCR = Polymerase Chain Reaction; CR, Control Region. IHC = Immunohistochemistry.

Immunohistochemistry ‘-, negative; +, 1–30% cell positivity; ++, 31–60% cell positiity; +++, >61% cell positivity. n = nuclear; c = cytoplasmic.

### T-Antigen and β-catenin co-localize to the nucleus in colon cancer cells and co-immunoprecipitate *in vitro*

**Figure 3:**
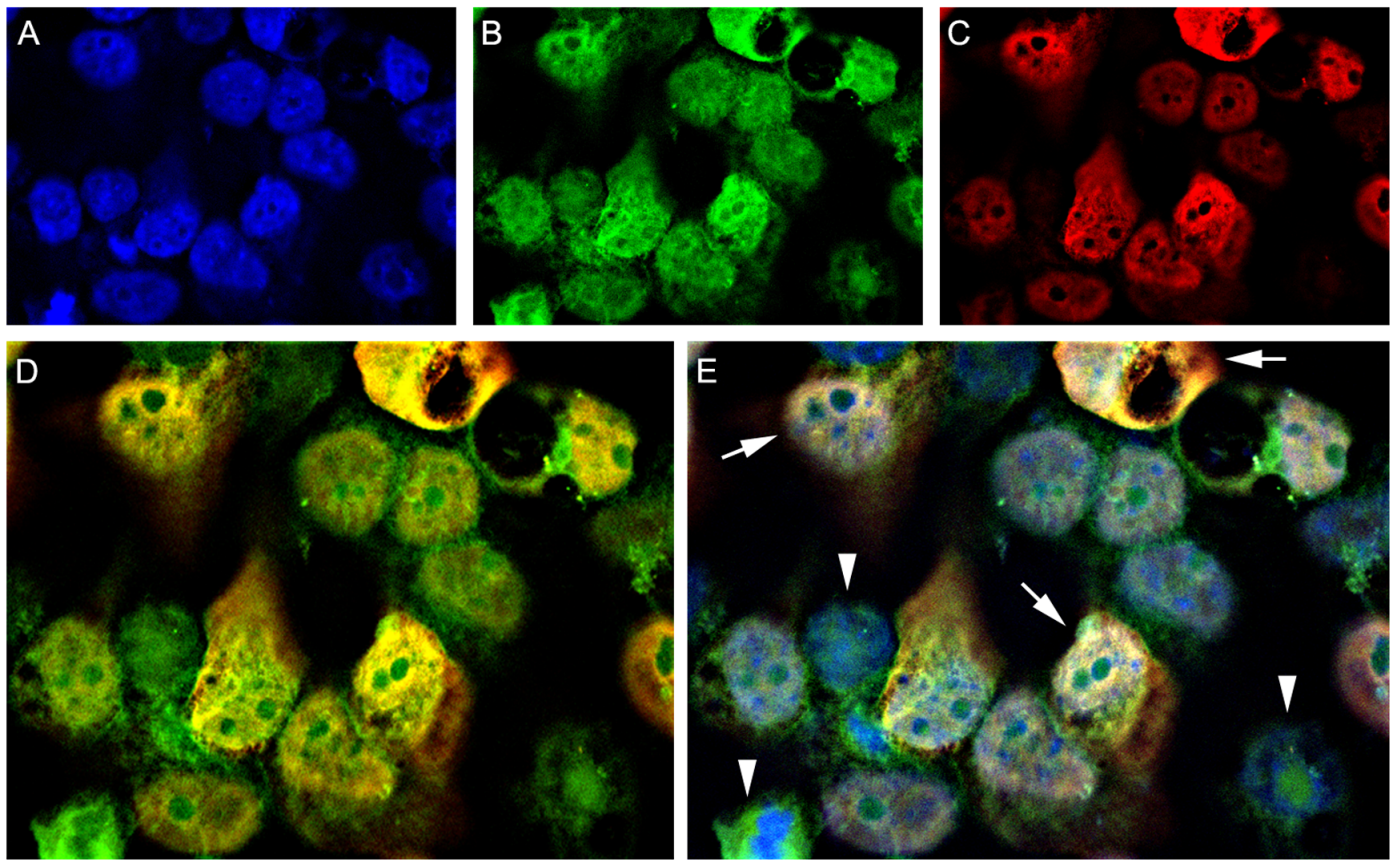
T-Antigen and β-catenin co-localization *in vitro*. Double labeling immunocytochemistry for β-catenin (Panel B, Fluorescein), and T-Antigen (Panel C, Rhodamine), performed in HTC116 colon cancer cells transiently transfected with T-Antigen show the co-localization of both proteins in the nuclei of the majority of cells (Panel E, arrows), while in non-transfected cells in which there is no expression of T-Antigen, β-catenin remains exclusively in the cytoplasm (Panel E, arrowheads).

Previous studies suggest that T-Antigen and β-catenin interact directly altering the normal cytoplasmic subcellular localization of β-catenin. In order to corroborate these findings *in vitro* and to determine whether T-Antigen and β-catenin are present in the same cellular compartment in colon cancer cells, we performed dual labeling immunofluorescence for these two proteins in the HCT116 colon cancer cell line transiently transfected with T-Antigen. We found that cells expressing the oncoprotein show a strong nuclear expression of β-catenin (Figure 3D–E, arrows), while in cells that did not get transfected and do not express T-Antigen, β-catenin remains in the cytoplasm (Figure 3D–E, arrowheads).

**Figure 4:**
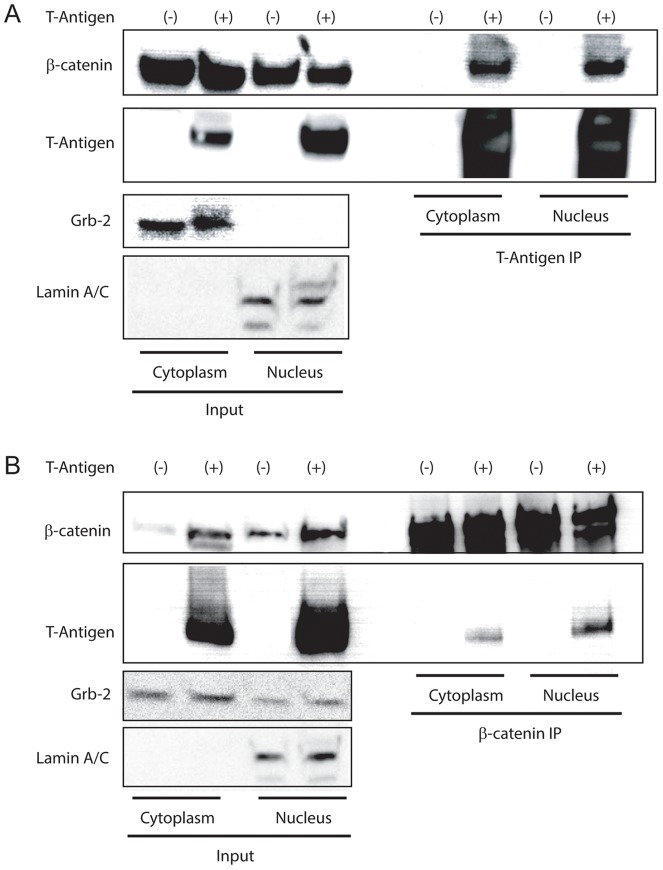
Interaction between T-Antigen and β-catenin in colon cancer cells. HCT116 cells transfected with T-Antigen or empty vector were fractionated to isolate nuclear and cytoplasmic compartments and immunoprecipitated with an antibody against T-Antigen (A) or β-catenin (B). T-Antigen pulls down β-catenin in the cytoplasm (top panel, lane 6) and in the nucleus (top panel, lane 8). The inverse experiment (IP β-catenin) confirms the interaction between T-Antigen and β-catenin in the cytoplasm (bottom panel, lane 6) and nucleus (bottom panel, lane 8).

To further define the subcellular location of the physical interaction between T-Antigen and β-catenin, we performed a co-immunoprecipiation on nuclear and cytoplasmic extracts of HCT116 cells transfected with a T-Antigen expression vector or empty vector as controls. Nuclear and cytoplasmic lysates were incubated with an anti-T-Antigen antibody and the immunocomplexes were analyzed via Western blot with antibodies against T-Antigen and β-catenin. T-Antigen immunoprecipitates from the nucleus and cytoplasm show co-precipitation of β-catenin in both compartments (Figure 4A). We confirmed this interaction in the inverse experiment (β-catenin IP, T-Antigen Western blot- Figure 4B). The specificity of nuclear and cytoplasmic fractions was confirmed by probing input lanes for Grb-2 (cytoplasmic) and lamin A/C (nuclear).

### T-Antigen activates TCF-dependent transcription

**Figure 5:**
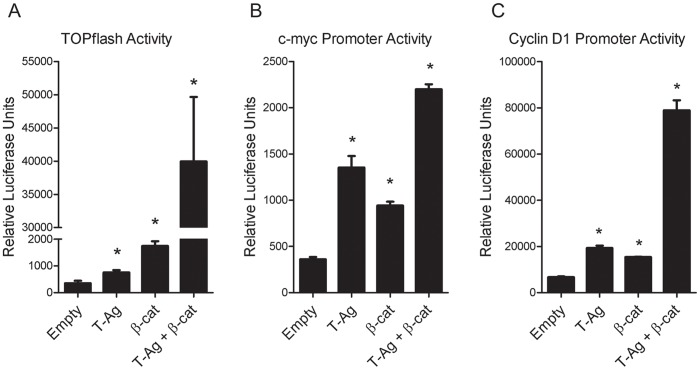
T-Antigen interacts with β-catenin to activate β-catenin target gene promoters. HCT116 cells were co-transfected with the Wnt pathway reporter plasmid TOPflash construct and T-Antigen, β-catenin, or both. Cells transfected with T-Antigen or β-catenin alone increased TOPflash activity approximately 2- to 5-fold over baseline. Co-transfection with both T-Antigen and β-catenin resulted in 113-fold increase in TOPflash activity. Values are normalized to FOPflash (baseline) groups (Panel A). To measure activation of specific β-catenin targets, similar experiments were conducted using luciferase constructs containing the c-Myc (Panel B) or Cyclin D1 (Panel C) promoters. Expression of T-Antigen significantly increases c-Myc and Cyclin D1 promoter activity in HCT116 cells. This effect was potentiated by co-transfection with a β-catenin expression vector, resulting in a four- to ten-fold increase in promoter activity. **p≤0.01; ***p≤0.001.

Following confirmation that T-Antigen and β-catenin co-localize and interact in the nucleus, we investigated the effects of this interaction on the activation of β-catenin target genes. We utilized the TOPflash reporter construct to measure changes in β-catenin/TCF-mediated transcription. The TOPflash construct contains 16 TCF binding elements (TBEs) driving expression of the luciferase gene. HCT116 cells were transfected with TOPflash and either T-Antigen, β-catenin, or both T-Antigen and β-catenin. Luminescence was measured at 24 hours post-transfection. All groups were separately transfected with FOPflash to measure baseline luminescence. T-Antigen or β-catenin alone significantly activated TOPflash by 2-fold and 5-fold over empty vector control, respectively. Strikingly, cells expressing T-Antigen and exogenous β-catenin showed a 113-fold increase in TOPflash activity over baseline (Figure 5A, p≤0.001). These data demonstrate the ability of T-Antigen and β-catenin to synergistically activate the promoters of β-catenin target genes. Values are normalized to FOPflash-transfected cells.

### T-Antigen increases promoter activity of c-Myc and Cyclin D1

To determine activation of specific β-catenin target genes that control cellular transcription and cell cycle regulation by T-Antigen by T-Antigen, we chose to study two known proto-oncogene targets of β-catenin mediated transcription, c-Myc and Cyclin D1. We co-transfected HCT116 cells with luciferase constructs containing either the c-Myc promoter or the Cyclin D1 promoter along with either T-Antigen, β-catenin or both, and performed promoter reporter activation assays. Our findings demonstrate that T-Antigen and β-catenin individually increased c-Myc (Figure 5B, p≤0.01) and Cyclin D1 (Figure 5C, p≤0.001) promoter activity, however co-transfection with T-Antigen and β-catenin causes a potentiated increase in c-Myc (6.1-fold) and Cyclin D1 (11.7-fold) promoter activity over empty vector controls.

## Discussion

The human neurotropic virus JCV is the established opportunistic agent of the fatal demyelinating disease Progressive Multifocal Leukoencephalopathy, frequently seen in patients with AIDS [5], and in recent years in patients undergoing immunomodulatory therapies for auto-immune diseases [6]. On the other hand, the oncogenicity of JCV in animal models and its transforming abilities *in vitro* are well recognized, despite the fact that its role in the development of human tumors was debated for some time. However, during the last decade, an increasing number of studies provide clear evidence for the role of JCV in brain and gastrointestinal tract tumorigenesis. More than 6 independent laboratories have detected JCV and studied the mechanisms behind its oncogenic role in colon cancer [10], [23]–[30]. Interestingly, patients with advanced colorectal neoplasia have significantly higher JCV antibody titers than healthy controls, suggesting an association between viral load or reactivation and risk of transformation [54]. The JCV protein large T-Antigen is a potent transforming oncoprotein, structurally and effectively similar to SV40 T-Antigen, and is expressed in ∼90% of cancers of the esophagus [11] and ∼35–65% of colon cancer cases [10], [55]. Furthermore, T-Antigen is associated with p53 expression and chromosomal instability in colon cancer cases [55] and has been shown to interact with other cell signaling proteins, most notably β-catenin [10], [49]. However, the effects of this interaction are not fully understood. Herein we demonstrate a relationship between JCV T-Antigen and β-catenin in human colon cancer cases and we elucidate the effects of this interaction *in vitro* on the activation of the β-catenin target genes c-Myc and Cyclin D1.

We show that T-Antigen and β-catenin co-localize in the nucleus of colon cancer cells *in vitro* and interact to enhance the activation of the β-catenin target genes c-Myc and Cyclin D1. This discovery suggests that T-Antigen mediates β-catenin translocation to the nucleus and transcription of downstream target genes.

There have been many different, but not mutually exclusive hypotheses about the downstream effects of the interaction between β-catenin and T-Antigen. Ricciardiello *et al* showed that JCV infection causes chromosomal instability *in vitro* [56]. Gan and Khalili first showed that T-Antigen binds and stabilizes β-catenin through the N-terminal end of β-catenin in a medulloblastoma cell line and protects it from trypsin degradation in medulloblastoma and glioblastoma cell lines [41], [52], indicating that T-Antigen can inhibit interaction of β-catenin with other proteins. Later, the same group showed that T-Antigen stabilization of β-catenin results in an interaction between β-catenin and activated Rac1 at the cell membrane [49]. Activation of Rac1 results in a number of pro-oncogenic signals including activation of the MAPK, JNK, NF-κB, and PI3K pathways. We have shown here that T-Antigen and β-catenin physically interact in the nuclei of colon cancer cells and that this stabilization leads to the activation of TCF-dependent transcription as measured by TOPflash activity and c-Myc and Cyclin D1 promoter activation. In addition to those mechanisms demonstrated previously, our work suggests a role for enhanced activation of Wnt pathway target genes by T-Antigen in conjunction with β-catenin. c-Myc and Cyclin D1 are both powerful proto-oncogenes with similar downstream effects. c-Myc has a number of putative targets, including genes involved in cell cycle control, apoptosis, DNA metabolism and dynamics, energy metabolism and macromolecular synthesis [57]. Cyclin D1 is responsible for cell cycle progression in the transition from G0/G1 to S phase and is overexpressed in parathyroid adenoma, centrocytic lymphoma, breast cancer, squamous cell carcinoma, and esophageal carcinoma [58]. Thus, enhanced activation of these genes in colon cancer through the expression of T-Antigen could result in unchecked cell cycle progression, a high proliferation rate, and ultimately a more malignant phenotype. Other noteworthy targets of Wnt signaling include MMP-7 [59], which enhances migration and invasion [60], the normally dormant anti-apoptotic protein Survivin [61], which is reactivated in colon cancer [62], and vascular endothelial growth factor (VEGF), which is important in promoting tumor growth through angiogenesis [63]. Thus, enhanced activation of Wnt pathway target genes by T-Antigen could result in a number of oncogenic effects such as evasion of apoptosis and enhanced metastatic potential. In support of the theory that T-Antigen expression could increase metastatic potential, Link *et al* showed that JCV-positive colon cancer cases are more likely to metastasize to the liver; this effect was shown to be T-Antigen-dependent *in vitro* [27]. Aberrant activation of Wnt target genes has the potential to drive the progression of cancer as most, if not all β-catenin target genes enhance the growth, development, invasion, and/or metastasis of cancer.

Increased β-catenin target gene activation by T-Antigen in the absence of Wnt activation may proceed by a number of different mechanisms. T-Antigen can block or sterically hinder the phosphorylation of β-catenin [41], [49], thus allowing the accumulation of free β-catenin in the cytoplasm and its translocation to the nucleus. A second possibility hinges on the nuclear localization of T-Antigen; since T-Antigen is often found at high levels in the nucleus of tumor and transformed cells due to its classical nuclear localization signal (NLS), T-Antigen may bind β-catenin in the cytoplasm and facilitate the movement of β-catenin to the nucleus. A third, and possibly concomitant option is that T-Antigen and β-catenin localize to the nucleus separately, and once there, interact to prevent the export of β-catenin. This would retain active β-catenin in the nucleus and allow the constant activation of target genes. These mechanisms are not mutually exclusive and may be acting together to promote a neoplastic phenotype. Thus, more work is required to determine the specific effect of T-Antigen on β-catenin stability and the subsequent activation of transcription.

JCV and its oncoprotein T-Antigen have long been a focus of investigation to elucidate its transformative properties and to determine its role in the development of cancer. Many groups have demonstrated the presence of T-Antigen in tumors of the GI tract and CNS, with a few explanatory mechanisms of transformation. Here we provide evidence for an association between T-Antigen and β-catenin in colon cancer and an enhanced transcriptional activation of Wnt pathway target genes by β-catenin in the presence of T-Antigen. These data suggest that the mechanism underlying the proliferation of cells expressing JCV T-Antigen involves the activation of Wnt pathway target genes and therefore may provide important therapeutic or prognostic value.
